# The Curse of Normalization

**DOI:** 10.1002/cfg.192

**Published:** 2002-08

**Authors:** Olaf Wolkenhauer, Carla Möller-Levet, Fatima Sanchez-Cabo

**Affiliations:** 1 Department of Biomolecular Sciences Control Systems Centre UMIST Manchester M60 1QD UK; 2 Department of Electrical Engineering Control Systems Centre UMIST Manchester M60 1QD UK

## Abstract

Despite its enormous promise to further our understanding of cellular processes involved in the regulation of gene expression, microarray technology generates data for which statistical pre-processing has become a necessity before any interpretation of data can begin. The process by which we distinguish (and remove) non-biological variation from biological variation is called *normalization*. With a multitude of experimental designs, techniques and technologies influencing the acquisition of data, numerous approaches to normalization have been proposed in the literature. The purpose of this short review is not to add to the many suggestions that have been made, but to discuss some of the difficulties we encounter when analysing microarray data.


**Comparative and Functional Genomics**
*Comp Funct Genom* 2002; **3**: 375–379.
Published online in Wiley InterScience (www.interscience.wiley.com). **DOI:** 10.1002/cfg.192

**Conference Review**

### The curse of normalization

Olaf Wolkenhauer,[1,2]* Carla Möller-Levet[2] and Fatima Sanchez-Cabo[1]

[1] *Department of Biomolecular Sciences, Control Systems Centre, UMIST, Manchester M60 1QD, UK*
[2] *Department of Electrical Engineering, Control Systems Centre, UMIST, Manchester M60 1QD, UK*

**Correspondence to: Olaf Wolkenhauer, Department of Biomolecular Sciences, Department of Electrical Engineering, Control Systems Centre, UMIST, Manchester M60 1QD, UK. E-mail: o.wolkenhauer@umist.ac.uk*

Received: 31 May 2002
Accepted: 12 June 2002

### Abstract

**Despite its enormous promise to further our understanding of cellular processes involved in the regulation of gene expression, microarray technology generates data for which statistical pre-processing has become a necessity before any interpretation of data can begin. The process by which we distinguish (and remove) non-biological variation from biological variation is called *normalization*. With a multitude of experimental designs, techniques and technologies influencing the acquisition of data, numerous approaches to normalization have been proposed in the literature. The purpose of this short review is not to add to the many suggestions that have been made, but to discuss some of the difficulties we encounter when analysing microarray data.** **Copyright © 2002 John Wiley & Sons, Ltd.**

**Keywords:** microarrays; bioinformatics; normalization; singular value decomposition

### Introduction

Figure 1 outlines some of issues dealt with in microarray bioinformatics. An important point is that 'data analysis' is distinct from 'data management' and that the analysis of data begins already with the acquisition of images. At this early stage decisions have to be made about how poor or empty spots are dealt with. Statistics extracted from the images form the basis for these decisions, which naturally will influence the 'downstream' analysis (e.g. clustering and classification). While 'poor spots' should naturally be excluded from the analysis, in the absence of replicates, a missing value in a time series may force the analyst to ignore the gene associated with a missing data point.

Removing noise, detecting outliers and filling in missing values are problems for which solutions are developed in statistics. The basis for any such approach is to use information in the data set to fill in gaps or remove undesirable effects. In the absence of sufficiently reliable information (e.g. through replicates or large time series), data analysis becomes an *art*, enjoyed by statisticians, dreaded by the rest.

### What is normal in normalization?

Table 1 summarizes *one* approach to the normalization of array data, divided into four (five) sequential steps. There are many different approaches to normalization, all of which depend on the technology employed and the experiments considered. Serving as an example, the table does not claim completeness (e.g. housekeeping genes and spiked controls are not considered) and we refer to the list of selected references for further reading [1–7].

The problem we wish to illustrate here is that through normalization we correct for possible non-biological variation but thereby can also reduce the informative content of the data. This does not mean one should not normalize; however, one pays a price for correcting something that is not completely known. Let us consider the following sequential steps in the normalization process, summarized in Table 1.

Copyright © 2002 John Wiley & Sons, Ltd.

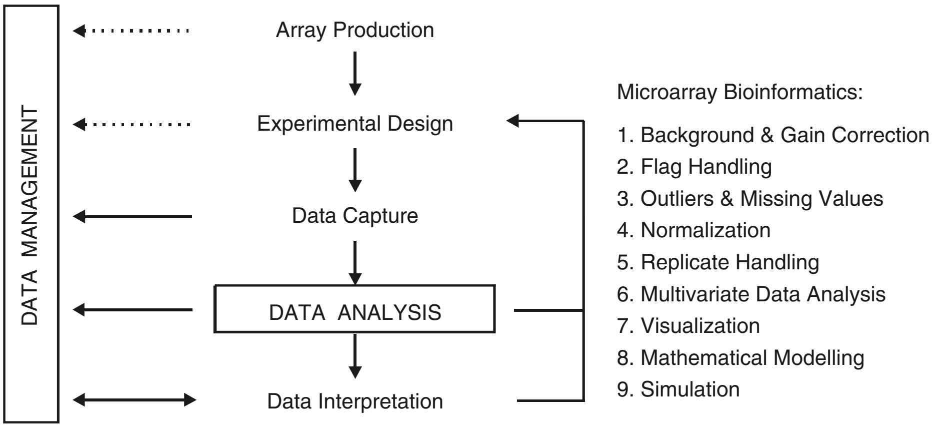

**Figure 1.** Microarray bioinformatics begins with the acquisition of data and their management (storage of experimental data and information associated with the experiment). Data analysis, visualization and interpretation are the aim of the whole process, one that can be daunting at times

Table 1. An example for the normalization of DNA microarray data

| Normalization | Variation | Options | Implementation | |
|---|---|---|---|---|
| Background subtraction | | Background subtraction for all genes $i = 1, \ldots$ | $s_i = s_i - bs_i$<br>$r_i = r_i - br_i$ | |
| | | No subtraction | $s_i = s_i$<br>$r_i = r_i$ | |
| Dye correction | Dye effects | Lowess function | $A_i = 1/2 \cdot (\log_2 s_i + \log_2 r_i)$<br>$M_i = \log_2(s_i/r_i)$ | $s_i = s_i$<br>$r_i = r_i k(A_i)$ |
| | | Linear regression (RNA vs. RNA) | From scatter plot:<br>$s = m \cdot r + a$ | $s_i = (s_i - a)/m$<br>$r_i = r_i$ |
| | | Gain correction (DNA vs. RNA) | $m_i = br_i/bs_i$ | $s_i = s_i \cdot m_i$<br>$r_i = r_i$ |
| Per-spot normalization | Array and gene effects | | $p_i = s_i/r_i$ | |
| Within-array normalization | Gene effects | Median (50% percentile) | $q_i = p_i/med_i(p_i)$ | |
| | | Percentiles (e.g. 25%, 75%) | $q_i = p_i/perc_i(p_i)$ | |
| DNA vs. RNA time course experiments: (after averaging replicates for every time point.) | | | | |
| Across-arrays normalization | Array effects | Against all arrays | $e_i = q_i/med_j(q_j)$ for all genes $i$, for all arrays $j = 1, \ldots$ | |
| | | Against arrays in $J$ | $e_i = q_i/med_j(q_j)$ for all genes $i$, and for arrays $j$ e $J$ | |

The values $s$, $r$ refer to intensities in the signal and reference channel, respectively; $bs$, $br$ denote the intensity of the background measured for both channels. Note that gain correction may not be appropriate for experiments with swapped dyes and if the relationship existing between background intensities of both channels is different to the one of signal intensities.

### Background subtraction and gain correction

Information about background intensities can be used in different ways to normalize the data set. Some authors [1] suggest subtracting the background intensity from the signal intensity in both channels (we hereafter refer to one channel as the reference and the other as the signal). This process makes the assumption that background and

Copyright © 2002 John Wiley & Sons, Ltd. 

signal intensities are additive. The background can also be useful to correct for different gain settings during scanning of the array to accommodate for the different labelling efficiencies of the dyes. If background intensities in both channels differ, we assume the existence of external factors that modify the measurements of intensities and potentially mask biological variation. We calculate (for each spot) a constant, $m_i$, to equal the background intensities for both channels and, in consequence, to remove non-biological variation due to the interaction between dye and sample (see Table 1).

In an RNA vs. RNA microarray experiment we expect the quantity of initial mRNA to be the same for both labelled samples. In terms of intensities, this means that the total intensity measured for both channels should be very similar. In a scatter plot (reference against signal intensities) we would therefore expect the data points to be distributed along a 45° line (i.e. a line with a slope equal to 1). We can fit a regression line through the points in the scatter plot and subsequently transform the data to align them along the ideal line, with a slope equal to 1. The calculations are shown in Table 1 for a useful illustration of this process, see [5]. We note in passing that for non-linear relationships between both channels, the use of the Lowess function approach is more appropriate than linear regression [7].

### Per spot normalization

With the handling of liquids in minute quantities and measurements at relatively small scales, many experimental variations can occur. The most obvious experimental errors (e.g. liquid remains on the array surface) are visible in the scanned image. These spatial effects usually affect both channels. We account for this artificial variation by dividing the signal intensity of one channel by the other. With this simple operation we are at the same time comparing the relative intensity of both channels, something that is the principal aim of RNA vs. RNA experiments.

### Within-array normalization

The hybridization efficiency may vary from gene to gene spotted in the microarray. With the normalization within an array we balance this variation. Relating each intensity ratio to one and the same number, we ensure that these are comparable. This number should be representative of the overall intensity in the array. In practice, this number is chosen to be a percentile of intensities in the array (or subregions of the array). Which percentile is chosen depends on the number of genes that are expected to be differentially expressed, e.g. in RNA vs. RNA experiments we use the median (50% percentile) of the intensity ratios because we assume the same proportion of genes being up- and downregulated.

### Replicate handling

If we are dealing with replicated experiments, we require a value that summarizes the biological information collected by the different experimental replicates. One value that fulfils this criterion is the average. In the particular case of a time series experiment, every new value obtained through averaging is going to represent a time point. As for genes within an array, in time course experiments we require the same gene to be comparable across arrays. This is what in Table 1 is referred to as *normalization across arrays*. Following normalization, we consider the obtained expression levels in log-space.

### Other ways to normalize: SVD and ANOVA

The biggest challenge the biologist faces in the analysis of array data is to identify a suitable normalization method for his/her experiment. We can illustrate the effect of non-biological variation using singular value decomposition (SVD) of the gene expression matrix (GEM). The SVD of the GEM allows us to describe every gene expression profile as the linear combination of some 'fundamental' or 'principal' patterns of variation. Figure 2 illustrates principal patterns, which are also referred to as 'eigengenes', for a data set that is not normalized. Figure 3 shows the SVD on the same data set but after normalization. We notice that the second eigengene for the non-normalized data disappears in the SVD of the normalized data, while the other principal patterns

Copyright © 2002 John Wiley & Sons, Ltd. 

remain. This suggests that the second eigengene does represent the non-biological variation removed by the normalization process described in previous sections. An alternative approach to normalization would therefore be the 'filtering' of the gene expression profiles by subtracting the eigengene corresponding to the 'noise' (unwanted variation).

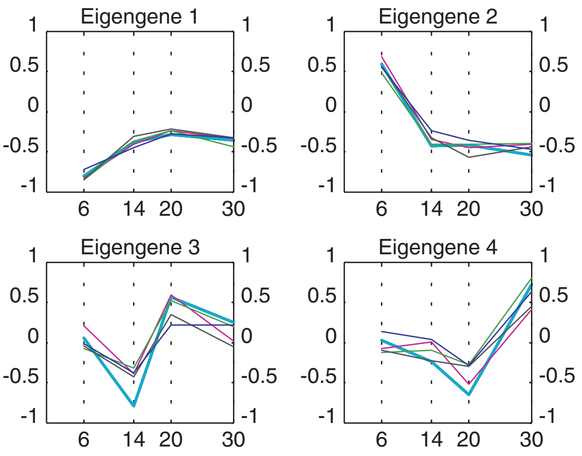

**Figure 2.** The decomposition of the gene expression matrix shows four 'principal patterns' (bold lines) that occur in the data. Each graph shows four original expression profiles that correlate well with the principal or eigengene

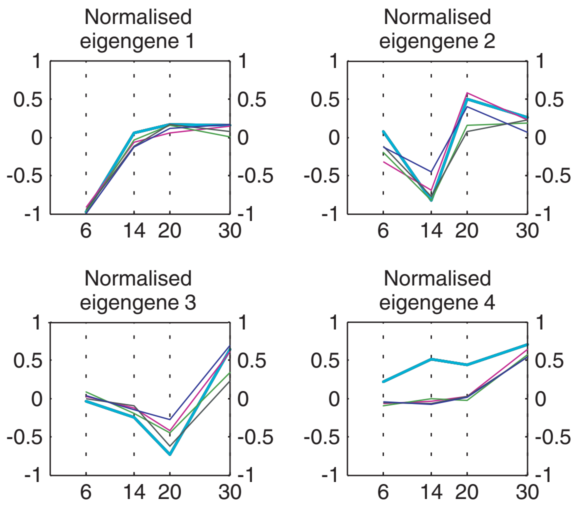

**Figure 3.** The SVD of the normalized data set shows that the second eigengene in Figure 2 has vanished. This suggests that eigengene 2 captures the non-biological variation we removed through the normalization process described in Table 1

ANOVA (analysis of variance) is a popular statistical approach to consider sources variation in array data. ANOVA tries to achieve a valid estimator to understand the variability of a data set, detecting all the possible sources of variation.

We can define the main sources of variation in a microarray experiment and summarize them in one equation. As described in [3,4], we could express every realization of the experiment as a combination of all these factors:

$$\log(y_{ijkg}) = \mu + A_i + D_j + V_k + G_g + (AG)_{ig} + (DV)_{jk} + \varepsilon_{ijkg}$$

where $y_{ijkg}$ = measurement of the gene $g$ in the array $i$ for the dye $j$ and the variety $k$; $\mu$ = overall average signal; $A_i$ = effect of the $i$th array; $D_j$ = effect of the $j$th dye; $V_k$ = effect of the $k$th variety; $G_g$ = effect of the $g$th gene; $(AG)_{ig}$ = a particular gene in a particular array; $(DV)_{jk}$ = interaction between the $k$th variety and the $j$th dye; and $\varepsilon_{ijkg}$ = error term. Using ANOVA we can obtain an estimator for every one of these variables and use the expression above to correct the signal intensities.

### Conclusion

The diversity of methods on offer and the many considerations necessary in the process of normalization can be daunting for anyone for whom the primary focus of array data analysis is their interpretation. Microarray data analysis therefore serves as a good example of the need for close interdisciplinary collaborations between biologists and data analysts.

### Acknowledgements

We would like to acknowledge the support of the Bacterial Microarray Group at St George's Hospital, London. They not only provided the authors with good quality data sets but also proved to be outstanding collaborators. Their guidance and support helped the authors to better understand the production of microarrays. One conclusion from this work on data analysis is that such close interdisciplinary links are vital for the analysis and interpretation of array data. We are also grateful to The Wellcome Trust for their support in bringing the UMIST and St. George's groups together.

Copyright © 2002 John Wiley & Sons, Ltd. 

### References

1. Herzel H, Beule D, Kielbasa S, *et al.* 2001. Extracting information from cDNA arrays. *CHAOS* **11**: 98–107.
2. Huber W, von Heydebreck A, Sültmann H, Poustka A, Vingron M. 2002. Variance stabilization applied to microarray data calibration and to the quantification of differential expression. *Bioinformatics* (in press).
3. Kerr K, Churchill GA. 2001. Experimental design for gene expression analysis. *Biostatistics* **2**: 183–201.
4. Kerr K, Martin M, Churchill GA. 2000. Analysis of variance for gene expression microarray data. *J Comput Bio* **7**: 819–837.
5. Quackenbush J. 2001. Computational Analysis of Microarray Data. *Nature Rev Genet* **2**: 418–425.
6. Tsodikov A, Szabo A, Jones D. 2002. Adjustments and measures of differential expression for microarray data. *Bioinformatics* **18**(2): 251–260.
7. Yang YH, Dudoit S, Luu P, *et al.* 2001. Normalization for Microarray Data: **http://stat-www.berkeley.edu/users/terry/zarray/Html/normspie.html.**

Copyright © 2002 John Wiley & Sons, Ltd. 
